# Health Anxiety and Its Correlates in the General Chinese Population During the COVID-19 Epidemic

**DOI:** 10.3389/fpsyt.2021.743409

**Published:** 2021-11-22

**Authors:** Jia Luo, Pengchong Wang, Zhanjiang Li, Wenwen Cao, Huan Liu, Limin Meng, Jing Sun

**Affiliations:** ^1^The National Clinical Research Center for Mental Disorders & Beijing Key Laboratory of Mental Disorders, Beijing Anding Hospital, Capital Medical University, Beijing, China; ^2^Advanced Innovation Center for Human Brain Protection, Capital Medical University, Beijing, China; ^3^Menzies Health Institute Queensland and School of Medicine and Dentistry, Griffith University, Brisbane, QLD, Australia

**Keywords:** COVID-19, epidemic, health anxiety, depression, chronic disease

## Abstract

**Background:** The novel coronavirus disease 2019 (COVID-19) pandemic has had an adverse impact on the mental health of the general population. This study aimed to investigate the prevalence and correlates of health anxiety (HA) in the general Chinese population to inform psychological interventions in COVID-19-affected areas.

**Methods:** We conducted an online survey of the general population in mainland China between 6 and 17 February 2020 (*N* = 1,450, 69.79% female; mean age = 37.5 ± 9.1 years). The Whiteley Index-7 (WI-7), COVID-19 knowledge quiz (CKQ), Generalised Anxiety Disorder scale (GAD-7), Patient Health Questionnaire Depression Scale (PHQ-9), and socio-demographic information were surveyed using the Questionnaire-Star program.

**Results:** The prevalence of HA, depression and anxiety were 47.3, 31.3, and 35.7%, respectively. The WI-7 score showed a significant association with age, education level, income, occupation, chronic disease and daily time focused on COVID-19. On binary logistic regression analysis, individuals with masters or higher qualification degree [odds ratio (OR) = 0.632)], older age *(OR* = 0.981), 2-4 h daily time focused on COVID-19 (*OR* = 0.684), healthcare workers (*OR* = 0.749, *p* = 0.046) and those with more COVID-19 related knowledge (*OR* = 0.785) showed a significantly negative association with HA. Chronic disease (*OR* = 1.962), depression (*OR* = 1.05) and anxiety (*OR* = 1.228) were significant risk factors for HA.

**Conclusions:** HA was highly prevalent among the general population during the early stages of the COVID-19 outbreak. More than two-fifths of the respondents had obvious HA. Chronic disease, depression and anxiety were risk factors for HA; psychological interventions offered during the pandemic should pay particular attention to these individuals.

## Introduction

The World Health Organization (WHO) announced a Public Health Emergency of International Concern (PHEIC) on 30 January 2020. The ongoing COVID-19 pandemic is a cause of psychological stress. The early phase of the COVID-19 epidemic induced considerable depression and anxiety among the general population. A study conducted in China documented moderate to severe depressive symptoms in 16.5% of the population, moderate to severe anxiety symptoms in 28.8% of the population, and moderate to severe stress levels in 8.1% of the population (1). Furthermore, studies have shown that the large amount of publicity about this disease can induce health anxiety (HA) even in medically healthy people (2, 3).

HA is excessive worrying about one's health, which is inappropriate or excessive relative to the actual health status (4). It occurs in a continuous spectrum (5) that ranges from a slight focus on health to strong health-related fears and preoccupation with beliefs about health (also referred to as illness anxiety disorder or hypochondriasis). Severe HA may cause significant distress, functional impairment and excessive healthcare utilisation (4, 6).

People with high HA misinterpret bodily sensations and overestimate the risk of serious illness. These individuals tend to experience higher levels of fear of being infected, so HA can contribute to the psychological consequences of the current pandemic. Previous studies also suggest that HA is the predictor for virus anxiety, COVID-19 distress, COVID-19 stress syndrome and post-traumatic stress symptoms (7–10). In addition, individuals with HA believe that their health is threatened, so they engage in body-oriented checking behaviours or avoidance. During a pandemic, some people with high HA may contribute to the health system overload by seeking reassurance from physicians, while others may be reluctant to seek medical care due to concerns that hospitals are sources of transmission. Thus, addressing HA plays an important role in the success of public health strategies to counter pandemics (2). However, the prevalence of HA among the general population during the COVID-19 pandemic is not well characterised. Most surveys of psychological distress during the COVID-19 pandemic have not used questionnaires specific for health anxiety, such as the Health Anxiety Inventory (HAI) or the Whiteley Index (WI); for example, the nationwide large-scale survey of psychological distress in the Chinese general population the COVID-19 epidemic (11). Current knowledge shows that the prevalence of HA among Chinese college students during the pandemic was 24.3% (12), and one-third (30.3%) of students had elevated HA in the United States (13). To the best of our knowledge, the prevalence of HA among the general population has not yet been established during the tumultuous period of the COVID-19 pandemic.

Social-demographic and health behaviour factors have a great influence on HA. Previous studies have found that age, occupational status, physical illness and smoking predicted HA (14, 15). Excessive sadness and disability can also lead to HA. Research from Turkey showed that female gender, accompanying chronic disease and previous psychiatric history were risk factors for HA during a COVID-19 outbreak (16). Health risk communication and social media use were important in predicting HA among college students (12).

Despite this evidence, there is a paucity of epidemiological data and information on the factors that influence HA pertaining to the COVID-19 outbreak among the general population in mainland China. People from different cultures have different responses to COVID-19, so the cultural context will affect the incidence of HA as well as the factors that influence it.

To fill these research gaps, we conducted a cross-sectional nationwide online survey of the general population in mainland China during the first month of the COVID-19 epidemic. As discussed above, the extant literature implicates several candidate anxiety and distress in conjunction with socio-demographic factors of health anxiety responding to the threat of a COVID-19 pandemic. Accordingly, we hypothesise that the incidence of health anxiety will increase during the tumultuous time of the COVID-19 epidemic especially in the early stages. In addition, health anxiety may be related to general anxiety and depression level when the effects of educational level, health condition, and network use are accounted for. The purpose of the study was to assess the prevalence of HA and the potentially associated general anxiety and depression level together with socio-demographic and health behaviour factors at a time when the COVID-19 epidemic was rapidly spreading, especially during the tumultuous early phase of the COVID-19 outbreak. The results of the study will inform the development and implementing relevant mental health promotion policies to cope with the challenges brought by the pandemic efficiently and effectively.

## Materials and Methods

### Sample and Data Collection

Researchers sent the online survey link through social medial networks in China, and participants completed the survey through the secure online questionnaire link hosted by WJX (www.wjx.cn) using a mobile phone or desktop browser. The online questionnaire consisted of questions that covered several aspects: HA, general anxiety, depression, socio-demographic variables, past medical history, and COVID-19-related knowledge.

Participation was volunteer based, and all participants were informed about the anonymity of their responses and the measures taken to protect their privacy. Before the survey, participants were asked to read a statement about how their data was collected, saved and used, and then sign consent form to participate in the study. The study was approved by the Ethics Committee of the Beijing Anding Hospital, Capital Medical University [Ethics No. (2020) Scientific Research No. (9)].

A total of 1,506 subjects participated in the survey. Of these, 56 were excluded because of invalid responses or participant duplication (as assessed from the participants' IP address and demographic information). Finally, data pertaining to 1,450 (96.3%) respondents were included in the analysis.

### Survey Instruments

#### The Whiteley Index-7

The WI is one of the most widely used instruments for assessment of HA (17). The WI-7 comprises seven items in a dichotomised response format (yes/no) for each item. The total score ranges from 0 to 7, with higher scores indicating greater HA. The Chinese version of the WI-7 has been shown to exhibit satisfactory internal consistency (Cronbach's α = 0.73) and stable test-retest reliability (18). **The Chinese WI-7 demonstrated moderate reliability (Cronbach's** **α** **=** **0.699) in this study**. Conradt et al. (19) suggested that a cut-off score of 1/2 yielded the best balance of sensitivity and specificity for screening purposes. To identify more individuals with potential HA, 1/2 were used as a cut-off score in the present study.

#### The 7-Item Generalised Anxiety Disorder Scale

The 7-item Generalised Anxiety Disorder Scale (GAD-7) has been widely used to assess anxiety disorders (20). It contains seven items, each of which is scored on a scale of 0–3; the total score ranges from 0 to 21, with higher scores indicating greater anxiety symptoms. The GAD-7 is effective for the assessment of anxiety in the general population (21). The Chinese version of the GAD-7 was shown to exhibit satisfactory reliability and validity (Cronbach's α = 0. 93); use of **6 as the cut-off score for anxiety disorder screening was associated with a sensitivity of 0.91 and specificity of 0.71** (22).

#### The 9-Item Patient Health Questionnaire Depression Scale

The 9-item Patient Health Questionnaire Depression Scale (PHQ-9) was included in the study to assess depressive symptoms (23). The instrument contains nine items; the total score ranges from 0 to 27, with higher scores indicating greater depressive symptoms. The PHQ-9 is an effective tool for measuring depression in the general population (24). The Chinese version of the PHQ-9 has satisfactory reliability and validity (Cronbach's α = 0.892); scores of 6, 12, and 15 are used as **cut-off points for mild, moderate and severe depression** (25).

### The COVID-19 Knowledge Quiz

The COVID-19 Knowledge Quiz (CKQ) is an eight-item single choice measure of knowledge about COVID-19. Participants' responses are scored as 0 (incorrect) or 1 (correct); the total score ranges from 0 to 8, with higher scores indicating more accurate knowledge about COVID-19 and the 2019–2020 outbreak. The quiz items were similar to the Zika Facts Quiz (ZFQ) (3). The CKQ items were based on information published by the Chinese Center for Disease Control and Prevention on their COVID-19 virus-related webpage (26). In the present study, item-total Pearson's correlation analysis shows that each item significantly correlated with the total score (*r*: 0.106~0.574, all significant with *p*-value < 0.001).

### Data Analysis

The statistical package IBM SPSS 26.0 was used for data analysis. Independent-sample *t*-tests and one-way ANOVA tests were used to compare the WI-7 scores among different demographic groups; LSD-tests were used for *post hoc* analysis. Independent-sample *t*-tests and Chi-squared tests were used to assess differences in continuous and categorical variables between individuals with and without HA, respectively. We used binary logistic regression to determine the effects of COVID-19-related factors on HA symptoms among the samples; odds ratio (*OR*) with 95% confidence intervals (*CI*) were calculated to determine the strength of association. Chi-squared tests were used to identify the associations between socio-demographic variables and HA. Two-tailed *p*-values <0.05 were considered indicative of statistical significance.

## Results

### Participant Characteristics and WI-7 Score Differences Among Demographic Groups

The mean age of the participants was 37.5 ± 9.1 years; 69.79% were female. Among the respondents, 25.59% were healthcare workers, 15.72% were suffering a chronic physical disease, 28.83% were without partner support (single, divorced or widowed). The prevalence of HA, depression and anxiety was 47.3, 31.3, and 35.7%, respectively.

We conducted one-way ANOVA and independent *t*-tests to analyse differences in HA among different socio-demographic groups according to age, gender, partner support, work, chronic disease, daily focus time (DFT), education level and gross monthly income. If any of these variables showed significant difference, these factors will be included in the stepwise regression analysis as confounding factors.

Across the age groups (18–24, 25–34, 35–44, 45–54, and 55–65 years) a significant univariate effect was found [*F*_(4, 1, 449)_ = 5.92, *p* <0.001)]: individuals aged 55–65 years (1.354 ± 1.535) and 45–54 years (1.443 ± 1.562) showed significant lower HA than the younger groups (18–24 years: 2.047 ± 1.564; 25–34 years: 1.984 ± 1.781; 35–44 years: 1.881 ± 1.865) (*t* ≥ 0.8289, *p* ≤ 0.022). In the gender groups, there was no significant HA between female (1.830 ± 1.767) and male participants (1.797 ± 1.756) (*t* = 0.329, *p* = 0.742). Individuals without partner support (1.947 ± 1.805) did not show significantly higher HA than those with partner support (1.770 ± 1.744) (*t* = 1.729, *p* = 0.084). Healthcare workers (e.g., doctors, psychologists, physiotherapists, nurses; 1.580 ± 1.670) reported significant lower HA compared to all other participants (1.903 ± 1.787) (*t* = 3.054, *p* = 0.002). Participants with chronic diseases (2.57 ± 1.998) reported significantly higher HA than those without a chronic disease (1.68 ± 1.680) (*t* = 6.414, *p* < 0.001).

When considering DFT on COVID-19-related information online, one-way ANOVA testing revealed a significant univariate effect in the DFT groups (<2 h, 2–4 h, >4 h) [*F*_(2, 149)_ = 12.299, *p* < 0.001). Participants who spent greater than 4 h (2.159 ± 1.930) showed higher HA than those who spent less than 2 h (1.63 ± 1.657, *t* = 4.822, *p* < 0.001) and those spending 2–4 h focused on COVID-19 (1.735 ± 1.677, *t* = 3.528, *p* < 0.001).

With respect to education level, there was no significant difference between subjects with a Masters or higher degree (1.858 ± 1.787), a Bachelor's degree (1.896 ± 1.791), and a junior college or lower degree (1.630 ± 1.670) [*F*_(2, 1, 449)_ = 2.808, *p* = 0.061]. With respect to gross monthly income (GMI), those with GMI > 10,000¥ (1.653 ± 1.716) showed lower HA than those with 5,000–10,000¥ GMI (1.936 ± 1.782, *t* = −2.510, *p* = 0.012) and <5,000¥ GMI (1.907 ± 1.789, *t* = −2.286, *p* = 0.022) [*F*_2, 1449_ = 3.986, *p* = 0.019].

### Demographic and Clinical Correlates of Health Anxiety

Based on the WI-7 screening cut-off score (1/2), the study population was divided into a HA group (HA, *n* = 686, 47.3%) and a non-HA group (NHA, *n* = 764, 52.7%). Within the HA group, 70.11% were females; the average age was 36.4 ± 8.6 years. HA showed a significant association with age, education level, healthcare worker, chronic disease, DFT, CKQ score, GAD-7 score, PHQ-9 score (Chi-squared test or independent *t*-test). The demographic characteristics of the participants are shown in Table 1.

**Table 1:** Group difference between HA and NHA in demographic and clinic variables.

**Variables**	**Total** **(*n* = 1,450)**	**HA** **(*n* = 686)**	**NHA** **(*n* = 764)**	***t/X2***	***P***
Age	37.5 ± 9.1	36.4 ± 8.9	38.5 ± 9.2	−4.445	<0.001
Female: Male	1,012/438	481/205	531/233	0.065	0.799
Partner support (Yes: No)	1,073/418	475/211	562/202	3.309	0.069
Education Junior college or below	402	199	203	9.243	0.01
Bachelor	691	343	348		
Master or above	357	144	213		
Healthcare worker (Yes: No)	317/1,079	150/536	221/543	9.465	0.002
Chronic disease (Yes: No)	228/1,222	143/543	85/679	25.769	<0.001
DFT <2 h	603	267	336	15.045	<0.01
2–4 h	407	177	230		
>4 h	440	242	198		
GMI <5,000¥	463	231	232	7.527	0.023
5,000–10,000¥	439	221	218		
>10,000¥	548	234	314		
CKQ	6.27 ± 0.809	6.15 ± 0.796	6.38 ± 0.808	−5.304	<0.001
GAD-7	4.495 ± 4.045	6.185 ± 4.364	2.976 ± 3.016	16.112	<0.001
PHQ-9	4.367 ± 4.455	5.945 ± 4.910	2.950 ± 3.435	13.313	<0.001
WI-7	1.820 ± 1.763	3.344 ± 1.373	0.452 ± 0.498	52.190	<0.001

*GAD-7, the 7-item Generalized Anxiety Disorder Scale; PHQ-9, the 9-item Patient Health Questionnaire Depression Scale; DFT, Daily focus time on COVID-19; CKQ: COVID-19 Knowledge Quiz; GMI, gross monthly income; HA, health anxiety group; NHA, non-health anxiety group; WI-Total, Whiteley Index-7 total score*.

### WI-7 Item Frequency Distribution Analysis

In the overall sample, the item with the highest positive rate (percentage of respondents with score = 1) was Item 6: “worry about getting a disease that was brought to your attention (e.g., on TV, radio, newspapers, or by someone you know)” (45.7%), followed by Item 2: “worry a lot about your health” (38.6%) and Item 3: “hard for you to believe the doctor when he or she tells you there is nothing to worry about” (27.7%). The item with the lowest positive rate was “think there is something seriously wrong with your body” (7.4%). For each of the seven items, the HA group showed a higher percentage of positive response (score = 1) than the NHA group. Within the HA group, apart from Items 2, 3, and 6, the participants also showed a higher percentage of positive responses (score = 1) for Item 4: “often worry about the possibility that you have a serious illness” (71.5%) (Table 2).

**Table 2:** The frequency distribution of the 7-item Whiteley Index.

**WI-7 items (English)**	**Score** **=** **1 percentage (%)**		
	**HA** **(*n* = 686)**	**NHA** **(*n* = 764)**	**Total** **(*n* = 1,450)**
1. Do you think there is something seriously wrong with your body?	21.8	1.0	7.4
2. Do you worry a lot about your health?	84.9	17.8	38.6
3. Is it hard for you to believe the doctor when he or she tells you there is nothing to worry about?	47.4	18.9	27.7
4. Do you often worry about the possibility that you have a serious illness?	71.5	6.2	26.4
5. Are you bothered by many different pains and aches?	40.3	5.8	16.5
6. If a disease is brought to your attention (e.g., On TV, radio, the newspapers, or by someone you know), do you worry about getting it yourself?	87.3	27.1	45.7
7. Do you find that you are bothered by many different symptoms?	52.1	5.1	19.7

*HA, health anxiety group; NHA, non-health anxiety group*.

*Score = 1 means response is “yes.”*

### Predictors of Health Anxiety

One-way ANOVA and t-tests were utilised to explore the differences in the difference between HA and Non-HA group in demographic groups, if there are differences in HA and non-HA group in any of these demographic factors these factors were controlled for in the stepwise multiple logistic regression analysis. In order to establish a predictor model, the binary variable of the WI group (HA = 1, NHA = 0) was used as the dependent variable, while GAD-7 and PHQ-9 scores, chronic disease, DFT, and CKQ were selected as independent variables and age, gender, partner support, education level, healthcare worker, and GMI as confounding factors, using a stepwise method [probability of stepwise: entry = 0.05, removal = 0.1]. In hierarchical logistic regression, (1) For the first step, the socio-demographic variables are included in the logistic regression; (2) For the second step, COVID-19-related internet use and knowledge factors were added into the logistic regression; (3) for the third step, anxiety and depression were included in the model. According to the Hosmer-Lemeshow test, the final model's Chi-square value was 7.821 (*p* = 0.451 > 0.2), which showed a good fit of the model with the original data. The overall percentage correct was 69.9%, which indicated that the model showed a moderate predictive accuracy.

As shown in Table 3, in the final model (third step analysis), older age (*OR* = 0.981), CKQ (*OR* = 0.785), Masters or higher degree (*OR* = 0.632), healthcare worker (*OR* = 0.749) and 2–4 h DFT (*OR* = 0.684) showed a significant association with lower risk of HA. Chronic disease (*OR* = 1.962), PHQ-9 score (*OR* = 1.05) and GAD-7 score (*OR* = 1.228) were significantly associated with a higher risk of HA (Table 3).

**Table 3:** Hierarchical logistic regression analysis of HA related risk and protect factors.

	**First step**				**Second step**				**Third step**			
	**B**	**OR**	**95% C.I**.	***P***	**B**	**OR**	**95% C.I**.	***P***	**B**	**OR**	**95% C.I**.	***P***
**Demographic variables**												
Age	−0.034	0.966	(0.953, 0.980)	<0.001	−0.033	0.968	(0.954, 0.982)	<0.001	−0.019	0.981	(0.966, 0.996)	0.014
**Gender**												
Male	−0.069	0.933	(0.740, 1.177)	0.558	−0.036	0.964	(0.762, 1.220)	0.763	0.226	1.253	(0.970, 1.619)	0.084
**Education level**												
Junior college or below	Ref			0.056	Ref			0.175	Ref			0.024
Bachelor	−0.061	0.94	(0.720, 1.228)	0.652	−0.012	0.988	(0.752, 1.298)	0.933	−0.102	0.903	(0.674, 1.210)	0.494
Master or above	−0.354	0.702	(0.51, 0.967)	0.03	−0.258	0.773	(0.556, 1.073)	0.124	−0.459	0.632	(0.442, 0.902)	0.011
**Partner support**												
Support with partner	0.083	1.086	(0.829, 1.424)	0.549	0.108	1.114	(0.846, 1.466)	0.442	0.06	1.062	(0.784, 1.439)	0.697
**GIM**												
>10,000¥	Ref			0.243	Ref			0.183	Ref			0.518
<5,000¥	0.085	1.088	(0.827, 1.433)	0.547	0.055	1.057	(0.799, 1.398)	0.7	−0.008	0.992	(0.733, 1.342)	0.957
5,000¥-10,000¥	0.223	1.25	(0.962, 1.623)	0.094	0.24	1.272	(0.976, 1.658)	0.076	0.145	1.156	(0.867, 1.541)	0.323
Healthcare worker	−0.345	0.708	(0.550, 0.091)	0.007	−0.32	0.726	(0.563, 0.937)	0.014	−0.289	0.749	(0.568, 0.988)	0.041
Chronic disease	0.832	2.298	(1.700, 3.107)	<0.001	0.873	2.394	(1.762, 3.253)	<0.001	0.674	1.962	(1.410, 2.730)	<0.001
**COVID-19 related factors**												
**DFT**												
>4 h					Ref			<0.001	Ref			0.055
<2 h					−0.462	0.63	(0.487, 0.815)	<0.001	−0.173	0.841	(0.635, 1.113)	0.226
2-4 h					−0.497	0.609	(0.458, 0.808)	0.001	−0.38	0.684	(0.502, 0.932)	0.016
CKQ					−0.321	0.726	(0.632, 0.833)	<0.001	−0.242	0.785	(0.677, 0.911)	0.001
**Clinical factors**												
GAD-7									0.205	1.228	(1.171, 1.287)	<0.001
PHQ-9									0.049	1.05	(1.008, 1.094)	0.018
Constant	1.12	3.064		0	3.309	27.362		0	1.179	3.251		0.043
R^2^	0.066				0.098				0.269			

*GAD-7, The 7-item Generalized Anxiety Disorder Scale; PHQ-9, The 9-item Patient Health Questionnaire Depression Scale; DFT, Daily focus time about COVID-19; GMI, gross monthly income; CKQ: COVID-19 Knowledge Quiz*.

## Discussion

Our study is the first to have investigated the HA of the general population in mainland China during the period from 6–17 February 2020, i.e., 1 week after the declaration of the COVID-19 epidemic as a PHEIC by the WHO. With the increased spread of COVID-19, there was widespread coverage of COVID-19-related information in traditional and social media. Of note, 47.3% of respondents reported HA; 31.3% of respondents suffered from mild to severe depression and 35.7% of respondents were experiencing anxiety. Our findings are consistent with those of another study conducted during the same period (1). This suggests that the COVID-19 pandemic has had a significant effect on the physical as well as the mental health of the general population. These findings indicate that addressing the mental health effects is an indispensable component of any public health intervention during the pandemic.

The present study focused on the incidence of HA during the COVID-19 outbreak. Using a cut-off score of 1/2, the prevalence of HA in our study population was 47.3%. As we know, the prevalence of HA in Chinese college students during the COVID-19 pandemic was 24.3%, which is lower than our result (12). This discrepancy may be related to the different pandemic stages. Our study data was collected from 6–17 February 2020, in which the epidemic was an uncertain and threatening situation, so more likely to trigger psychological symptoms. A recent meta-analysis showed wide variability in the reported rates of HA in the general population (range, 2.1–13.1%) (27). The reported lifetime prevalence of HA in Australia is approximately 5.7 %, while 3.4% of people were suffering from HA at the time of the interview (14). The incidence of HA in this study was significant higher than that in other studies (14, 27). This indicates that the COVID-19 outbreak is an important risk factor for HA. The ongoing COVID-19 pandemic is similar to the SARS pandemic in 2002, which was a traumatic experience for China (28). During the SARS outbreak, 5327 patients were infected and 348 people died from SARS in China (28). Hence, the COVID-19 outbreak evoked considerable concern by public health organisations; in addition, social media was flooded with alarming and psychologically distressing information about COVID-19. Previous studies have found a positive correlation between media consumption and anxiety (29, 30), which partly explains the elevated HA among the general population during the first month of the novel coronavirus outbreak. In addition, research and clinical observations suggest that people exhibit greater fear and anxiety associated with infection and illness during infectious disease outbreaks (31). In the present study, Item 6 of the WI-7 (If a disease is brought to your attention (e.g., on TV, radio, the newspapers, or by someone you know), do you worry about getting it yourself) had the highest positive rate.

The second objective of this study was to explore the correlates of HA among the general population during the COVID-19 epidemic. In the stepwise regression analysis, when depression and anxiety were added to the model, the total variances were significantly increased from 9.8 to 26.9% suggesting depression and anxiety were major and significant risk factors for HA; this is consistent with the results of previous studies (15, 32, 33). A previous study showed that the prevalence of HA among visitors to medical clinics is significant higher than that in the general population (33). Depression may be one of the most important determinants of HA (15). Depressed patients often ruminate and have negative automatic thoughts. Rumination about suffering a disease is liable to aggravate an individual's health-related anxiety (34). Anxiety is a key precursor to the development of HA. According to the cognitive behaviour model (35), HA is mainly associated with irrational health beliefs and catastrophic thinking; in addition, distorted cognition and HA are mainly regulated by anxiety and depression. Increased alertness caused by anxiety and people with existing chronic illness may further aggravate illness worries and irrational health beliefs, which is the most important manifestation of HA during pandemic of COVID-19 in China.

Although online media were the primary channel for the general public to access the latest health information in the initial stage of the COVID-19 epidemic (36), media consumption shows a positive correlation with anxiety (29, 30). In our study, a 2-4 h DFT, which reflect an adaptive information seeking behaviour, was significantly associated with a lower risk of HA. During the initial stage of the COVID-19 epidemic, DFT less than 2 h may limit individuals from knowing more about outbreak control and prevention information; but excessive or repeated online searches for epidemic-related information are anxiety-provoking. This is consistent with previous studies. A systematic review found a positive correlation of HA with an online search for health information (37). According to the cognitive-behavioural model, excessively focus on health information is not only a negative trigger, but also maintains an individual's HA in the long term. Searching for health information online is an important safety behaviour of individuals with high HA (38). Such individuals try to excessive or repeated seek online health information to reduce their anxiety. However, this may also expose them to more worrying or contradictory information (39). Furthermore, seeking online health information may further reinforce the anxiety of those who already have excessive anxiety about their health (40).

Other demographic characteristics related factors including age, education, gender and type of profession and knowledge of COVID-19 had confounding effects relating to the relationship of anxiety and depression and HA. In particular, individuals aged younger groups (aged 35–44, 25–34, and 18–24 years) than older age 55–65 years and 45–54 years showed significantly higher HA. In the stepwise regression model, older age could significantly predict a lower risk of HA. Although some researchers suggest that older adults may be especially prone to experiencing anxiety related to health because health problems threaten to reduce the control they have over their lives (41), a recent study showed seniors with relatively fewer health problems may experience reduced HA compared with other older adults and younger adults (42). In addition, younger age groups (18–29 and 30–44) experienced higher levels of depressive and generalised anxiety symptoms than older adults (45–59 and 60–85 years) during the COVID-19 pandemic (42). These findings suggest that we should not ignore the mental health of young people during the outbreak of infectious diseases. In our study, females did not report significantly higher HA than males, which in contrast to the study in Turkish society. In that study, female gender was found to be a risk factor for HA (16). The discrepancy may be related to the different ratios of male and female participants in this study. Among the respondents, 69.79% were female in our study; this gender ratio was similar to previous national studies (11). It may indirectly reflect that in China females are more concerned about their psychological condition.

To date, there is a paucity of studies about HA among healthcare workers. In a recent study, health-related workers reported less coronavirus-related anxiety (43). Healthcare workers have access to more accurate information about the virus and the pandemic, which explains their lower levels of anxiety. The results of greater COVID-19 knowledge correlated with less HA indirectly indicates the protective effect of health-related work against HA. In agreement with our results, previous studies have suggested that more accurate knowledge of Zika can significant predict less Zika-related anxiety (3).

According to the cognitive theory of HA, irrational health beliefs are at the core of HA; in addition, irrational health beliefs are primarily influenced by an individual's knowledge level and experience of illness (34). Most factors that protected against HA in the present study (CKQ, Master's or higher degree, healthcare work) also reflect the individual's knowledge level.

## Limitations of the Study

Our study is the first to have assessed the prevalence of HA in China and identified depression and anxiety were significant risk factors for HA using a large number of sample during COVID-19 pandemic time. However, the present study the study was not a true prevalence study due to the non-representative sample of an online study. Individuals who worry about their health are likely to spend more time seeking health information online (37). Hence, these individuals are more likely to self select themselves to take part in the survey. In addition, the majority of the participants were female (*n* = 1,012, 69.79%) and most participants came from Beijing (*n* = 603, 41.6%), which may have introduced an element of selection bias. Finally, the cross-sectional study design does not permit any causal inferences. Future studies should use large, representative sample and cohort or randomised controlled trail for measuring HA during the pandemic.

## Conclusions

This study revealed a high prevalence of HA among the general population during the early stage of the COVID-19 outbreak. Depression and anxiety were significant risk factors for HA. Other factors including age, COVID-19-related knowledge, education level and time focused on seeking COVID-19-related information online were influencing factors of HA.

Our findings will help inform public health interventions targeting high-risk groups and guide resource allocation for mental health interventions. Our research suggests that vulnerable groups should be given greater consideration, such as those with chronic disease, anxiety and depression. The government should provide these groups mental health support when necessary. To ease the HA of the general public, education is needed. Moreover, the government should publish epidemic-related information in a timely manner to the public accessing scientific information. It is also important to reduce the time people search for health-related information online. In general, these results should be interpreted in the context of the early stage of the COVID-19 epidemic in China.

## Data Availability Statement

The original contributions presented in the study are included in the article/supplementary material, further inquiries can be directed to the corresponding authors.

## Ethics Statement

The studies involving human participants were reviewed and approved by Beijing Anding Hospital, Capital Medical University, China. The patients/participants provided their written informed consent to participate in this study.

## Author Contributions

JL designed the study, performed literature searches, drafted the manuscript, and critically reviewed and revised the manuscript. PW performed literature searches, conducted the statistical analysis, and drafted the manuscript. WC, HL, and LM collected data. ZL conceptualised and designed the study and reviewed and revised the manuscript. JS conducted statistical analysis, critically reviewed, edited, and revised the manuscript. All authors approved the final manuscript as submitted and agree to be accountable for all aspects of the work.

## Funding

This work was supported by Beijing Municipal Administration of Hospitals Incubating Program (code: PX2018062; PX2020075) and Beijing Municipal Administration of Hospitals Clinical Medicine Development of Special Funding Support (code: XMLX202129 and ZYLX201815).

## Conflict of Interest

The authors declare that the research was conducted in the absence of any commercial or financial relationships that could be construed as a potential conflict of interest.

## Publisher's Note

All claims expressed in this article are solely those of the authors and do not necessarily represent those of their affiliated organizations, or those of the publisher, the editors and the reviewers. Any product that may be evaluated in this article, or claim that may be made by its manufacturer, is not guaranteed or endorsed by the publisher.
